# Cause of death and potentially avoidable deaths in Australian adults with intellectual disability using retrospective linked data

**DOI:** 10.1136/bmjopen-2016-013489

**Published:** 2017-02-02

**Authors:** Julian Trollor, Preeyaporn Srasuebkul, Han Xu, Sophie Howlett

**Affiliations:** Department of Developmental Disability Neuropsychiatry, School of Psychiatry, UNSW Australia, Sydney, New South Wales, Australia

**Keywords:** Intellectual disability, Mortality, Cause of death, Record linkage, Cohort studies

## Abstract

**Objectives:**

To investigate mortality and its causes in adults over the age of 20 years with intellectual disability (ID).

**Design, setting and participants:**

Retrospective population-based standardised mortality of the ID and Comparison cohorts. The ID cohort comprised 42 204 individuals who registered for disability services with ID as a primary or secondary diagnosis from 2005 to 2011 in New South Wales (NSW). The Comparison cohort was obtained from published deaths in NSW from the Australian Bureau of Statistics (ABS) from 2005 to 2011.

**Main outcome measures:**

We measured and compared Age Standardised Mortality Rate (ASMR), Comparative Mortality Figure (CMF), years of productive life lost (YPLL) and proportion of deaths with potentially avoidable causes in an ID cohort with an NSW general population cohort.

**Results:**

There were 19 362 adults in the ID cohort which experienced 732 (4%) deaths at a median age of 54 years. Age Standardised Mortality Rates increased with age for both cohorts. Overall comparative mortality figure was 1.3, but was substantially higher for the 20–44 (4.0) and 45–64 (2.3) age groups. YPLL was 137/1000 people in the ID cohort and 49 in the comparison cohort. Cause of death in ID cohort was dominated by respiratory, circulatory, neoplasm and nervous system. After recoding deaths previously attributed to the aetiology of the disability, 38% of deaths in the ID cohort and 17% in the comparison cohort were potentially avoidable.

**Conclusions:**

Adults with ID experience premature mortality and over-representation of potentially avoidable deaths. A national system of reporting of deaths in adults with ID is required. Inclusion in health policy and services development and in health promotion programmes is urgently required to address premature deaths and health inequalities for adults with ID.

**Strengths and limitations of this study**

- This study provides death statistics and details of potentially avoidable deaths in adults with intellectual disability (ID) who received disability services in New South Wales.
- The study provides evidence of mortality gaps in people with ID compared with the general population.
- The high proportion of potentially avoidable deaths for people with ID indicates an opportunity for the development of possible preventative strategies.
- Since the ID cohort is derived from registered users of disability services, people with mild ID may be under-represented
- These findings are not generalisable to everyone with ID.

## Introduction

Approximately 1.8% of the Australian population have an intellectual disability (ID).^1^ Compared with the general population, people with ID experience very poor mental and physical health status and substantial barriers to accessing quality health services.^2^ People with ID experience high rates of common disorders with known associations with mortality, including mental disorders, respiratory disorders, gastro-oesophageal reflux disease, epilepsy and obesity.^3–6^ Poor access to health services and an under-skilled medical workforce amplifies the potential for poor outcomes in this population group.

International research has demonstrated higher mortality rates in people with ID compared to the general population.^7–9^ Although life expectancy has increased over time,^8^ ^10–12^ premature death remains a feature for people with ID.^4^ ^7^ ^9–15^ Only limited examination of death in people with ID has been performed in Australia. Using a comprehensive service system database, Bittles *et al*^4^ examined life expectancy of people with ID, finding substantially reduced life expectancy and a negative association with severity of ID. However, there was no detailed examination of cause of death. A small population-based study of 40 deaths within a limited geographical area of New South Wales^13^ yielded a Standardised Mortality Ratio (SMR) of 4.9, and a predominance of deaths due to respiratory (35%), external (20%), cancer (17.5%) and heart disease (15%). The size of the study and the large number of deaths in people with more severe levels of disability limit the generalisability of these findings. The NSW Ombudsman reports on deaths in NSW disability accommodation services.^16^ ^17^ Although not representative of people with ID as a whole, this investigation highlights the value of close scrutiny of deaths data in vulnerable populations as it has identified preventable causes of death which have been the catalyst for improvements in policy and practice.^16^ However, the lack of routine collection and reporting of health outcomes and mortality data for people with ID is at odds with Australia's commitment under the United Nations Convention on the rights of persons with disabilities (UNCRPD).^18^

Some deaths are expected, particularly early in life in individuals with syndromal causes of ID associated with life-limiting medical conditions.^13^ However, this effect is minimised by accessing a broad range of disability service users of adult age. Comorbidity of ID with specific health conditions, poor access to appropriate healthcare, presence of excess cardiometabolic risk factors, polypharmacy, lifestyle choices, socioeconomic disadvantage and lack of self-advocacy and capacity to attend to one's health needs are likely to play a role in deaths of adults with ID.^8^ ^10^ ^19^ ^20^ However, the specific impact of many of these factors on mortality in this group awaits determination.

The NSW Department of Family and Community Services, Ageing Disability and Home Care collects a Disability Services Minimum Data Set (DS-MDS) related to all people with disability who are registered to receive funded disability services in NSW. This data set includes a large population of individuals who have ID as defined strictly in accordance with Diagnostic and Statistical Manual Fourth Edition (DSM IV) criteria. Similarities in ID services access between Australian states mean that this population approximates a nationally representative sample of people with ID who are in receipt of funded disability services. The linkage of the DS-MDS to mortality data enables the accurate identification of deaths and cause of deaths within a cohort of persons with ID within a defined period.

Previously, for the purposes of comparing death rates in NSW to international cohorts, we published age-standardised death rates in people with ID in 5-year age bands from 5 to 64 years of age^21^ according to the WHO standard age distribution.^22^ Consistent with international data, we observed an overall comparative mortality ratio of 2.55 in people with ID in NSW across this wide age range.^21^ The current study uses data standardised to the Australian population to extend these findings to examine other death indices and causes of death. We hypothesised that compared to the general adult population, the adults with ID would experience higher mortality rates, greater years of productive life lost (YPLL), different cause of death profiles and a higher proportion of potentially avoidable deaths.

## Methods

### Study populations

We used two distinct population data sets to define the ID and comparison cohorts. Overall, there were 42 243 registered clients with ID recorded as their primary or secondary disability within the DS-MDS from July 2005 to June 2012. Of these, 19 362 clients were aged 20 years and over at 1 July 2005 and were alive at 30 June 2005. Person years were calculated for each person with ID from 1 July 2005 and ended at 31 December 2011 or date of death (whichever occurred first), and these were grouped in 5-year age-bands (20 to 24, 25 to 29, etc). The comparison cohort was derived from the published Australian Bureau of Statistics (ABS) data (counts by 5-year age-bands) for the NSW general population from 2005 to 2011.^23^ Since the observation period for the ID cohort started from July 2005 (0.5 of a year), we multiplied the population counts in the ABS population data by 0.5 for that year only. Thus, the study spanned the period July 2005 to December 2011 inclusive. Similar to the ID cohort, we directly converted the population counts for the comparison cohort into person years.

### Record linkage

To observe mortality for individuals in the ID Cohort, de-identified linkage of the DS-MDS, and death records from the ABS and the NSW Registry of Birth Deaths and Marriages Death Records (RBDM) was performed by the NSW Centre for Health Record Linkage (CHeReL) based on a SLK581 Statistical Linkage Key. In accordance with best practice privacy preserving protocols, the linked unit record data were provided to the researchers after being stripped of personal identifiers.

### Outcome measures

*ID cohort:* Death events were derived from the NSW RBDM which records all deaths in NSW. To maximise our capacity to examine cause of death over the entire study period, we used two sources of cause of death information. ABS data with International Statistical Classification of Diseases and Related Health Problems, 10th Revision (ICD-10) coded causes of death were available from January 2005 to December 2007 only. The RBDM free text causes of death data were available from January 2005 to December 2011. We observed 732 deaths in the ID Cohort in the period from 1 July 2005 to 31 December 2011. Ninety-five (12.9%) of these deaths did not have a cause of death recorded in either the ABS or RBDM data sets, leaving 637 cases with cause of death data. Individuals who did not die or died after 31 December 2011 were assumed to be alive at 31 December 2011.

To derive coded cause of death data consistent with ABS conventions for the period for which it was unavailable from the ABS (January 2008 to December 2011), we used the standardised approach outlined in the ICD-10 Mortality Manual.^24^ After ensuring accuracy of coding by using the 155 cases with both RBDM free text cause of death and ABS for training purposes, a medical practitioner then used the RBDM free-text cause of deaths information to code the cause of death for the remaining deaths using the ICD-10 approach. Deaths so coded were pooled with the 155 ABS coded cases and assigned the term ‘ABS Convention’. This data set comprised the underlying cause of death for 637 cases coded according to ABS conventions, consisting of the ICD-10 coded cause of death available from ABS from June 2005 to December 2007 (155 deaths) and derived from RBDM from January 2008 to December 2011 (482 deaths).

Previous research has identified concerns about overshadowing of the true underlying cause of death^25^ and about coding errors, where deaths are attributed to the aetiology of the ID and therefore obscure the true underlying cause of death^26^ We therefore developed a revised underlying cause of death classification for those individuals for whom the aetiology of their ID had been coded as the underlying cause of death. To do this, we recoded the underlying cause of death for these cases to the cause of death which appeared immediately adjacent to the coded ID aetiology in reported sequence leading to death. A total of 102 deaths (49 deaths attributed to Down syndrome, 29 deaths attributed to cerebral palsy, 9 deaths attributed to other developmental disorders and 4 deaths attributed to chromosomal abnormalities) were thus ‘ID Revised’.

Potentially avoidable deaths are deaths from conditions that are preventable through individualised care and/or treatable through existing primary or hospital care for persons aged under 75 years and which are avoidable in the context of the present health system.^27^ We identified potentially avoidable deaths in the ID population under 75 years of age using underlying causes of death from both ‘ABS Convention’ and ‘ID Revised’ data.

*Comparison cohort:* We obtained NSW mortality information for the general population from the ABS website which publishes the number of deaths by age, for NSW and other states in Australia.^28^ ^29^ For causes of death by age group, we used information from the HealthStats NSW which publishes deaths for the general population in NSW by ICD10 category and by sex and age group. There was a negligible discrepancy between death counts obtained from these sources. We adjusted this published population and mortality data to make it directly comparable to our ID cohort. We multiplied the ABS number of deaths in 2005 by 0.5, for that year only to align data with the partial year available in the ID cohort. Health Stats NSW published data in the financial year format until 2010/2011, and therefore adjustment for 2005 was unnecessary. However, in order to make the Health Stats NSW data comparable to the deaths in the ID cohort for the period July 2010 to December 2011, we multiplied the number of deaths in the Health Stats NSW 2010/2011 data by 1.5. We restricted the age of individuals in the ID cohort to 20 years and over but the published data from Health Stats NSW had the category of 15–24 instead of 20–24; hence, we converted the frequency of deaths in the category by multiplying 0.5 for that age group only. For potentially avoidable deaths in NSW, yearly numbers of potentially avoidable deaths in NSW were available from the Health Stats NSW website.^27^ We adjusted the NSW potentially avoidable deaths data by multiplying the number of potentially avoidable deaths in 2011/2012 financial year by 0.5 so as to account for a partial financial year of 2011.

### Statistical analysis

Age-specific mortality rates were calculated as numbers of deaths of the people in each 5-year age-band from 20 years and over in the study period divided by total person years in that age range in the population. We calculated ASMRs for both the ID and comparison groups by matching the crude rates to the age profile of a ‘standard Australian population’ as at 30 June 2001, restricted to the 20 years and over age range and stratified by sex. The Comparative Mortality Figure (CMF) was calculated by dividing the age-standardised rate for ID groups by the comparison rate. In addition to the ASMR for 20 years and over, we also calculated ASMRs for specific age groups: 20–44 years; 45–64 years and 65 years and over.

The YPLL is an indicator for premature mortality and is the total number of years not lived by an individual who died before a prespecified age. To be consistent with the definition of potentially avoidable deaths, we considered people who died before 75 years of age as premature deaths. We calculated YPLL for each 5-year age band, for each cohort and separately for males and females. It was derived for each age group by multiplying the number of deaths by the difference between age 75 years and the median age in each age group, and summing the products obtained from each age group and dividing total potential years of life lost by the total population under 75 years of age.

We explored differences in underlying causes of death by ICD-10 chapter by ranking causes of death in both cohorts. For the ID cohort, we used our linked mortality information, while for the comparison cohort we used published NSW causes of death from Health Stats NSW.^28^ We also investigated the proportion of potentially avoidable deaths in the ID cohort and compared it with the NSW population. All causes of death were presented according to ICD-10 chapter. Owing to the small number of potentially avoidable deaths, we did not perform the direct standardised analysis but chose to compare the Incident Rate Ratio (IRR) of the potentially avoidable deaths in the comparison and ID cohorts using Poisson regression adjusting for age groups and ID status.

### Ethical approvals

The study was approved by the NSW Population and Health Services Committee (AU RED Reference: HREC/13/CIPHS/7; Cancer Institute NSW reference number: 2013/02/446).

## Results

The 19 362 individuals in the ID cohort accumulated 123 934 person years, had a median (IQR) age at July 2005 or at first contact with the service of 37 (27–48) years, and were predominantly males (10 813 individuals, 56% of the cohort). There were 732 deaths in people with ID, representing 4% of the cohort and equivalent to a crude death rate of 5.9 deaths per 1000 people per year. Of these, 435 (59%) deaths were of males giving a male to female ratio of deaths of 1.5. The median (IQR) age at death was 54 (42–64) years, and there was no significant difference (p=0.193) between median age at death for males (55 years) and females (52 years). Seventy-six per cent of deaths in the ID cohort occurred in people aged 64 years or younger. The comparison cohort experienced 305 050 deaths and accumulated 33 624 422 person years of exposure during the period of July 2005 to December 2011. This was equivalent to a crude death rate of 9.1 deaths per 1000 person years. In the comparison cohort, the estimated median (IQR) age at death was 81 (70–92) and the median age at death for males and females was 78 and 84, respectively. Fifty-one per cent of deaths in the comparison cohort were in males and 18% of death occurred in people aged 64 years or younger.

Table 1 details ASMRs for each age group with related CMFs. The ASMR in the whole ID cohort was 7.5 (95% CI 6.7 to 8.4) and in the comparison cohort was 5.7 (5.6 to 5.7), yielding a CMF of 1.3 (1.2 to 1.5). Higher age-specific mortality rates were apparent for both males and females with ID compared to the comparison cohort; ASMRs for all males and females in the ID cohort were 12.5 (10.5 to 14.5) and 8.6 (7.2 to 10.0), respectively, and 9.2 (9.2 to 9.3) and 6.6 (6.5 to 6.6) for the comparison cohort, respectively. Correspondingly, CMF for males with ID was 1.4 (1.1 to 1.6) and for females was 1.3 (1.1 to 1.6). Age-standardised mortality rates in the ID cohort increased across age bands, but ASMR differences with the comparison cohort led to substantial differences in CMFs across age bands. People with ID in the 20–44 years age category had four times the death rate of the comparison group CMF: 4.0; 95% CI 3.1 to 5.2). For people with ID in the age category of 65 years and over, there was no evidence to support excess deaths above the comparator (CMF: 1.0; 95% CI 0.8 to 1.2) (table 1). Age-specific mortality rates for the ID and comparison cohorts are shown in online supplementary table S1.

10.1136/bmjopen-2016-013489.supp1 Supplementary table 1

**Table 1:** ASMRs and CMFs and 95% CI

	ASMRs (per 1000 persons)						CMF (ID/comparison)		
	ID			Comparison					
Age group (years)	Male	Female	Total	Male	Female	Total	Male	Female	Total
20–44	2.9 (2.4 to 3.5)	2.9 (2.3 to 3.5)	2.9 (2.5 to 3.3)	1.0 (1.0 to 1.0)	0.5 (0.5 to 0.5)	0.7 (0.7 to 0.7)	3.0 (2.2 to 4.0)	6.1 (4.0 to 9.5)	4.0 (3.1 to 5.2)
45–64	9.0 (7.8 to 10.2)	7.5 (6.2 to 8.8)	8.3 (7.5 to 9.2)	4.5 (4.5 to 4.6)	2.7 (2.7 to 2.7)	3.6 (3.6 to 3.6)	2.0 (1.7 to 2.4)	2.8 (2.1 to 3.6)	2.3 (2.0 to 2.7)
65+	47.1 (35.8 to 58.4)	27.3 (20.0 to 34.6)	36.1 (29.8 to 42.5)	42.2 (42.0 to 42.5)	31.6 (31.5 to 31.8)	36.5 (36.3 to 36.6)	1.1 (0.9 to 1.4)	0.9 (0.7 to 1.1)	1.0 (0.8 to 1.2)
20+	12.5 (10.5 to 14.5)	8.6 (7.2 to 10.0)	7.5 (6.7 to 8.4)	9.2 (9.2 to 9.3)	6.6 (6.5 to 6.6)	5.7 (5.6 to 5.7)	1.4 (1.1 to 1.6)	1.3 (1.1 to 1.6)	1.3 (1.2 to 1.5)

ASMRs, Age Standardised Mortality Rates; CMFs, Comparative Mortality Figures; ID, intellectual disability.

The 732 deaths in the ID cohort resulted in 16 468 YPLL, of which 9673 YPLL occurred in males and 6795 in females. This yielded an overall YPLL rate in the ID cohort of 137 per 1000 people, but the YPLL rate was higher for males (144) than for females (128) with ID. The 305 050 deaths in the comparison cohort resulted in 1 488 636 YPLL, of which 951 455 YPLL occurred in males and 537 106 in females. The overall YPLL rate in the comparison cohort was 49 per 1000 people, and the YPLL rate was higher for males (49) than for females (35).

Causes of death were available for 637 of 732 deaths (87%) in the ID cohort and for 304 690 deaths in the comparison cohort. Table 2 presents the top 10 underlying causes of death by ICD-10 chapter, coded using ABS conventions and our revised method and compares these with the underlying causes of death in the comparison cohort. Of the 10 leading causes of death in the comparison cohort, 8 of the leading causes were shared with the ID cohort, regardless of the coding convention used for the latter. The dominant causes of death differed between the ID and comparison cohorts, as reflected in the ranking and proportion of deaths accounted for by specific ICD-10 chapter categories. Using ABS conventions, diseases of the circulatory system (18%), malignant neoplasms (18%), diseases of the nervous system (16%), as well as of the respiratory system (12%) and congenital malformations (11%), were the top five underlying causes of death and accounted for 75% of deaths in the ID cohort. Using the revised coding method, diseases of the respiratory system (20%), circulatory system (18%), malignant neoplasms (18%), diseases of the nervous system (13%) and injury and poisoning (6%) comprised the top five underlying causes of death and accounted for 75% of deaths in the ID cohort. In contrast, the leading five causes of death in the comparison cohort were diseases of the circulatory system (35%), malignant neoplasms (29%), diseases of the respiratory system (9%), injury and poisoning (5%) and mental and behavioural disorders (5%), which accounted for 81% of deaths. In an analysis based on ABS conventional coding of causes of death, we determined that the proportion of deaths from injury and poisoning in our subgroup of young adults with ID was low for both males (9%) and females (10%). In contrast, the proportion of such deaths in our comparison cohort was much higher and favoured males (52%) over females (35%; table 3).

**Table 2:** Top 10 underlying causes of death in people with and without ID

Underlying cause of death by ICD-10 chapter	ID ABS Convention		ID Revised		Comparison	
	Rank	Frequency (%)	Rank	Frequency (%)	Rank	Frequency (%)
All deaths						
Circulatory system	1	114 (18)	2	114 (18)	1	105 804 (35)
Neoplasms	1	114 (18)	3	113 (18)	2	88 540 (29)
Nervous system and sense organ disorders	3	103 (16)	4	80 (13)	6	11 573 (4)
Respiratory system	4	78 (12)	1	130 (20)	3	26 242 (9)
Congenital malformations, deformations and chromosomal abnormalities	5	72 (11)	8	28 (4)		
Injury and poisoning (incl. external causes of morbidity and mortality)	6	38 (6)	5	40 (6)	4	15 534 (5)
Digestive system	7	31 (5)	7	33 (5)	7	10 524 (3)
Mental and behavioural disorders	8	29 (5)	6	34 (5)	5	13 977 (5)
Endocrine, nutritional and metabolic diseases	9	16 (3)	10	16 (3)	8	10 535 (3)
Symptoms, signs and abnormal clinical and laboratory findings not elsewhere classified	10	14 (2)				
Genitourinary system			9	17 (3)	9	7493 (2)
Certain infectious and parasitic diseases					10	5235 (2)
Total		637		637		304 690
Potentially avoidable deaths	50 938 (17)					
Circulatory system	1	69 (11)	1	75 (12)		
Infections	2	32 (5)	2	60 (9)		
Cancer	3	31 (5)	3	32 (5)		
Other external causes of morbidity and mortality	4	28 (4)	4	30 (5)		
Respiratory system	5	16 (3)	5	16 (3)		
Total		199 (31)		240 (38)		

ID ABS, Intellectual Disability Australian Bureau of Statistics; ICD-10, International Classification of Diseases, 10th Revision.

**Table 3:** Top three leading causes of death and related proportions in the ID and the Comparison cohorts

Age group (years)	ID ABS		Comparison	
	Male	Female	Male	Female
20–44	Nervous system (27)	Nervous system (25)	Injury and poisoning (52)	Neoplasms (35)
	Respiratory (17)	Neoplasms (19)	Neoplasms (15)	Injury and poisoning (30)
	Circulatory system (14)	Congenital malformations (12)	Circulatory system (14)	Circulatory system (11)
45–64	Circulatory system (21)	Neoplasms (21)	Neoplasms (41)	Neoplasms (56)
	Neoplasms (18)	Congenital malformations (15)	Circulatory system (25)	Circulatory system (15)
	Nervous system (15)	Nervous system (13)	Injury and poisoning (11)	Injury and poisoning (6)
65+	Circulatory system (25)	Circulatory system (30)	Circulatory system (35)	Circulatory system (40)
	Respiratory system (20)	Neoplasms (29)	Neoplasms (31)	Neoplasms (22)
	Neoplasms (14)	Congenital malformations (12)	Respiratory (10)	Respiratory (9)

The top five potentially avoidable deaths in the ID cohort using ABS conventions and the ‘revised’ coding of deaths are also presented in table 2. Regardless of the coding method, circulatory system deaths and infections were the two leading causes of potentially avoidable deaths, followed by cancer. Potentially avoidable deaths using ABS conventions were substantially higher in the ID cohort (199 of 637, 31% of deaths) compared to the NSW population (17% of deaths).^27^ ^28^ The revised underlying cause of death classification in the ID cohort resulted in an even higher rate (38%) of potentially avoidable deaths.

**Figure 1:**
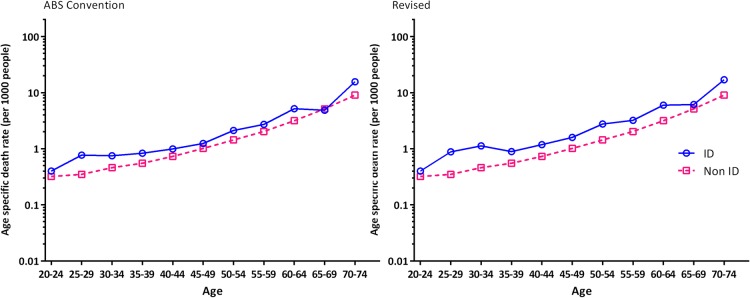
Age-specific avoidable death rates in people with and without ID. X Axis: Age group. Y Axis: Age-specific death rate (per 1000 persons). ABS, Australian Bureau of Statistics; ID, intellectual disability.

Age-specific potentially avoidable death rates for the ID and comparison cohorts are shown in figure 1 and in online supplementary table S2. People with ID had higher age-specific avoidable death rates than the comparison group for both ABS Convention and ‘Revised’ coding methods, *w*ith the latter being of slightly greater magnitude. After adjusting for age groups using Poisson regression, we found that compared to the comparison cohort, IRR was higher in the ID population using ABS convention coding (IRR 1.47; 95% CI 1.54 to 1.99; p<0.001), and even higher when we used the revised cause of death (IRR 1.77; 95% CI 1.56 to 2.01; p<0.001). We could not perform the analyses based on sex or causes as Health Stats NSW only published data of the potentially avoidable deaths by age groups.

10.1136/bmjopen-2016-013489.supp2 Supplementary table 2

## Discussion

This is the largest Australian study, and one of only a handful of studies worldwide which have investigated mortality and its causes in a representative sample of adult disability service users with ID. The experience of this population is of premature death, relative over-representation of deaths in young and middle-aged adults, and deaths from preventable causes.

Markers of premature death for people with ID in this cohort include predominance (76%) of deaths occurring prior to the age of 65 years, elevated ASMR (7.5 compared to 5.7 in the comparison cohort), a CMF of 1.3 and raised YPLL of 137 per 1000 people compared with 49 in the comparison cohort. Such figures reflect the stark and continuing health inequalities experienced by people with ID in Australia, and the lack of substantial progress in addressing these inequalities since initial publication of mortality data in Australians with ID more than a decade ago.^4^ ^13^

Similar to the general population, the overall ASMR for adult males with ID (12.5) was substantially higher than for adult females with ID (8.6). However, the impact of sex on ASMRs differed with age. In young and middle-aged adults, ASMRs of males and females with ID approximated one another, whereas in the corresponding age bands of the general population, ASMRs for males were approximately double those for females. Relative over-representation of deaths in females with ID has been previously documented in the international literature,^9^ ^11^ and possible explanations have been proposed including vulnerability to death conferred by specific interaction of sex with hormonal, genetic or mortality related risk factors, or other factors such as gender specific lifestyle factors or gender specific difficulty in accessing appropriate preventative healthcare including cancer screening. Our results suggest a further possible explanation. It is well known that deaths from injury and poisoning account for just under half (45%) of all young adult deaths in the general population and that the sex ratio of deaths from these causes strongly favours deaths in males.^30^ The low proportion of injury and poisoning deaths in males and females with ID compared with the general population suggests a relative under-representation of deaths in young males with ID (compared to the general population) as a driver of observed sex differences, rather than an over-representation of deaths in young females with ID.

Although the overall CMF for the adult ID cohort was only 1.3, the CMF in specific age groups revealed a substantially elevated CMFs of 4.0 in younger adults (20–44 years), moderately elevated CMF of 2.3 for middle-aged (45–64 years) adults and a CMF of 1.0 in older adults (65 years and over) with ID. Elevated CMFs in early adulthood may be driven by continuing effects from complex physical comorbidities associated with the aetiology of ID, whereas midlife mortality in people with ID most likely represents the impacts of premature age-related morbidities. A healthy survivor effect could explain the lack of over-representation of deaths in the elderly with ID. Future detailed analysis of cause of death profiles in different age groups will assist in deepening the understanding of the differential mortality rates at different life stages in people with ID.

Although there are similarities in the top 10 causes of death in people with and without ID, the proportions of deaths explained by specific cause of death differ between the groups. Compared to people without ID, people with ID have an over-representation of deaths related to diseases of the respiratory and nervous systems, as well as those of congenital and chromosomal origins and an under-representation of deaths due to age-dependent causes such as diseases of the circulatory system and neoplasms. Using the revised underlying cause of death coding, the order but not the composition of the top five underlying causes of death was changed, reflecting an emphasis on diseases of the respiratory and circulatory systems which was obscured by coding of the disability aetiology as the cause of death. Respiratory deaths are likely to be reflective of greater vulnerability to respiratory infections, including those that arise from feeding and swallowing difficulties.^17^ Deaths attributed to nervous system causes include those related to epilepsy, which is known to be over-represented in people with ID^31^ and in whom there may be an increased risk of sudden death.^32^

People with ID had much higher proportions of potentially avoidable deaths than people without ID. Potentially avoidable deaths in people with ID were dominated by deaths due to infections, diseases of the circulatory system, cancer and other external causes. The predicted ageing of the ID population with ID will most likely see increases in circulatory and neoplasm-related deaths unless specific preventative health strategies are implemented. Particularly stark is the large proportion of potentially avoidable deaths due to infections. Such deaths suggest that people with ID experience delays, difficulties or differences in accessing specific and effective interventions for infections. Medical assistance must be sought assertively in individuals who manifest symptoms, but this is made difficult as patients with ID may not readily report symptoms, and some providing direct care lack skills in early identification of relevant physical signs. Primary care providers should consider careful assessment, proactive treatment and close monitoring of progress if there are infections in this population.

Comprehensive strategies to mitigate risk of death due to respiratory causes have been recommended by the NSW Ombudsman.^16^ ^17^ Although some improvement to practice within disability services has been achieved, further work is required to ensure a systemic and interagency response to this issue, both within disability and health sectors. Critically, awareness raising and the implementation of specific education and targeted interventions in primary and specialist care settings are required. The implementation of the National Disability Insurance Scheme (NDIS) will bring increased choice and control for people with ID and their families. It is critical that the need for feeding and swallowing assessments, and awareness of vulnerability to infections and respiratory conditions are recognised, and that services are readily accessed and appropriately prioritised by people with ID and their carers under the NDIS.

This study has a number of implications for policy, practice and administration. Foremost, these data highlight the substantial and continuing health inequalities experienced by people with ID. In view of Australia's commitment to the UNCRPD,^18^ Australian governments are urged to focus attention on this population of need, and develop comprehensive responses which address the inadequacies of health policy, services and services access for this population. Strategies should be paired with regular reporting of health status and outcomes for people with ID, as occurs in other jurisdictions such as the UK. At the coal face, doctors should be equipped, through inclusion of minimum content in medical curricula nationwide, with knowledge, skills and attitudes which will assist improved access to quality health services in primary and specialist care. Capacity for coordinated care, including the mapping of service pathways and resources for people with ID, should be developed in each primary health network nationwide. Comprehensive and uniformly available specialist ID health services should be developed and made accessible to all people with ID. Nationwide, a range of health services currently funded by state government disability services are threatened with defunding in the roll-out of the NDIS and these should be transferred to health and expanded. Key performance indicators should be developed and collection of outcome measures stipulated for local health districts in relation to services provision to people with ID. Finally, this study demonstrates that cause of death can be better understood in people with ID if the aetiology of the disability is not coded as the underlying cause of death. The current convention obscures relevant and potentially avoidable causes of death for this population and should be formally revised.

Our study is not without limitations, as our ID cohort was derived from people who registered for disability services and our cohort only covers 7 years of data. Our ID cohort accounted for 0.6% of the NSW population in 2011, and people with mild ID may therefore be under-represented. Independent validation was not performed on our coding of deaths data for those deaths which were not previously coded by RBDM. However, a rigorous coding process was undertaken which mirrored that used for the national data set (ABS). However, the strengths of this study include the robust data linkage, report of underlying cause of death and potentially avoidable deaths, and that our sample is representative of disability service users with ID. Furthermore, our future work will expand the cohort size and duration of study, to allow examination of trends in death rates over time, and to enable a more detailed examination of the factors associated with premature death in people with ID.

## References

[1] Australia Institute of Health and Welfare. Disability in Australia: intellectual disability. Bulletin no 67*.* Canberra: AIHW, 2008.

[2] Trollor J Making mental health services accessible to people with an intellectual disability. Aust N Z J Psychiatry 2014;48:395–8. 10.1177/0004867414531628 24788903

[3] Beange H, McElduff A, Baker W Medical disorders of adults with mental retardation: a population study. Am J Ment Retard 1995;99:595–604. 7632427

[4] Bittles AH, Petterson BA, Sullivan SG The influence of intellectual disability on life expectancy. J Gerontol A Biolo Sci Med Sci 2002;57:M470–2. 10.1093/gerona/57.7.M470 12084811

[5] Lennox NG, Kerr MP Primary healthcare and people with an intellectual disability: the evidence base. J Intellect Disabil Res 1997;41(Pt 5):365–72. 10.1111/j.1365-2788.1997.tb00723.x 9373816

[6] Sutherland G, Couch MA, Iacono T Health issues for adults with developmental disability. Res Dev Disabil 2002;23:422–45. 10.1016/S0891-4222(02)00143-9 12426010

[7] Heslop P, Blair PS, Fleming P The Confidential Inquiry into premature deaths of people with intellectual disabilities in the UK: a population-based study. Lancet 2014;383:889–95. 10.1016/S0140-6736(13)62026-7 24332307

[8] Patja K, Iivanainen M, Vesala H Life expectancy of people with intellectual disability: a 35-year follow-up study. J Intellect Disabil Res 2000;44(Pt 5):591–9. 10.1046/j.1365-2788.2000.00280.x 11079356

[9] Tyrer F, Smith LK, McGrother CW Mortality in adults with moderate to profound intellectual disability: a population-based study. J Intellect Disabil Res 2007;51:520–7. 10.1111/j.1365-2788.2006.00918.x 17537165

[10] Coppus AMW People with intellectual disability: what do we know about adulthood and life expectancy? Dev Disabil Res Rev 2013;18:6–16. 10.1002/ddrr.1123 23949824

[11] Emerson E, Glover G, Hatton C Trends in age-standardised mortality rates and life expectancy of people with learning disabilities in Sheffield over a 33-year period. Tizard Learning Disability Review 2014;19:90–5. 10.1108/TLDR-01-2014-0003

[12] Janicki MP, Dalton AJ, Henderson CM Mortality and morbidity among older adults with intellectual disability: health services considerations. Disabil Rehabil 1999;21:284–94. 10.1080/096382899297710 10381241

[13] Durvasala S, Beange H, Baker W Mortality of people with intellectual disability in northern Sydney. J Intellect Dev Disabil 2002;27:255–64.

[14] Hollins S, Attard MT, von Fraunhofer N Mortality in people with learning disability: risks, causes, and death certification findings in London. Dev Med Child Neurol 1998;40:50–6. 9459217

[15] Tyrer F, McGrother C Cause-specific mortality and death certificate reporting in adults with moderate to profound intellectual disability. J Intellect Disabil Res 2009;53:898–904. 10.1111/j.1365-2788.2009.01201.x 19694898

[16] New South Wales Ombudsman. *Report of Reviewable Deaths in 2010 and 2011. Volume 2: Deaths of people with disabilities in care*. Sydney NSW: NSW Ombudsman, 2013: 21–60.

[17] New South Wales Ombudsman. *Report of Reviewable Deaths in 2012 and 2013. Volume 2: deaths of people with disability in residential care*. NSW Ombudsman, Level 24, 580 George Street, Sydney NSW 2000, 2015.

[18] United Nations. Convention on the rights of persons with disabilities. Geneva: United Nations, 2006.

[19] Haveman M, Heller T, Lee L Major health risks in aging persons with intellectual disabilities: an overview of recent studies. J Policy Pract Intellect Disabil 2010;7:59–69. 10.1111/j.1741-1130.2010.00248.x

[20] Pramyothin P, Khaodhiar L Metabolic syndrome with the atypical antipsychotics. Curr Opin Endocrinol Diabetes Obes 2010;17:460–6. 10.1097/MED.0b013e32833de61c 20717020

[21] Florio T, Trollor J Mortality among a cohort of persons with an intellectual disability in New South Wales, Australia. J Appl Res Intellect Disabil 2015;28:383–93. 10.1111/jar.12190 25994286

[22] Ahmad O, Boschi-Pinto C, Lopez A *Age standardization of rates: a new WHO standard. GPE Discussion Paper Series: No.31*. World Health Organization, 2001.

[23] Australian Bureau of Statistics. 3101.0—Australian Demographic Statistics, Sep 2014. Secondary 3101.0—Australian Demographic Statistics, Sep 2014 30 March 2015. http://www.abs.gov.au/AUSSTATS/abs@.nsf/DetailsPage/3101.0Sep%202014?OpenDocument

[24] Centres for Disease Control and Prevention. ICD-10 Mortality Manual 2a 2015 2015 2015 http://www.cdc.gov/nchs/nvss/instruction_manuals.htm

[25] Heslop P, Lauer E, Hoghton M Mortality in people with intellectual disabilities. J Appl Res Intellect Disabil 2015;28:367–72 10.1111/jar.12196 26011045

[26] Landes SD, Peek CW Death by mental retardation? The influence of ambiguity on death certificate coding error for adults with intellectual disability. J Intellect Disabil Res 2013;57(12):1183–90. 10.1111/j.1365-2788.2012.01614.x 22957894

[27] Centre for Epidemiology and Evidence. Potentially avoidable deaths. Secondary Potentially avoidable deaths. 28 August 2015. http://www.healthstats.nsw.gov.au/Indicator/bod_avodth/bod_avodth_age

[28] Australian Bureau of Statistics. 3303.0—Causes of Death, Australia, 2011. Secondary 3303.0—Causes of Death, Australia, 2011 30 March 2015. http://www.abs.gov.au/AUSSTATS/abs@.nsf/DetailsPage/3303.02011?OpenDocument

[29] Australian Bureau of Statistics. 3302.0—Deaths, Australia, 2013 Secondary 3302.0—Deaths, Australia, 2013 30 March 2015. http://www.abs.gov.au/AUSSTATS/abs@.nsf/DetailsPage/3302.02013?OpenDocument

[30] Centre for Epidemiology and Evidence. Health Statistics New South Wales. Secondary Health Statistics New South Wales. 17 March 2015. http://www.healthstats.nsw.gov.au

[31] Kerr M, Scheepers M, Arvio M, Guidelines Working G. Consensus guidelines into the management of epilepsy in adults with an intellectual disability. J Intellect Disabil Res 2009;53:687–94. 10.1111/j.1365-2788.2009.01182.x 19527434

[32] Young C, Shankar R, Palmer J Does intellectual disability increase sudden unexpected death in epilepsy (SUDEP) risk?. Seizure 2015;25:112–6. 10.1016/j.seizure.2014.10.001 25457453

