# Supplementary material for: Cause of death and potentially avoidable deaths in Australian adults with intellectual disability using retrospective linked data
Source: BMJ Open. 2017 Feb 2;7(2):e013489. doi: 10.1136/bmjopen-2016-013489 (PMC5306525; doi:10.1136/bmjopen-2016-013489)
Supplement: Supplementary table 1 [file bmjopen-2016-013489supp_table1.pdf]

Supplementary Table 1: Age and sex specific mortality rate per 1000 persons, in people with and without ID

Age group	Overall		Male		Female	
	ID	Comparison	ID	Comparison	ID	Comparison
20-24	1.92	0.43	2.34	0.61	1.37	0.24
25-29	2.53	0.49	3.23	0.69	1.70	0.29
30-34	3.49	0.64	3.30	0.90	3.73	0.37
35-39	2.79	0.83	2.62	1.10	3.00	0.56
40-44	3.74	1.21	3.15	1.55	4.52	0.88
45-49	5.53	1.88	5.63	2.34	5.41	1.43
50-54	6.51	2.85	7.21	3.57	5.64	2.15
55-59	9.73	4.30	10.23	5.47	9.05	3.18
60-64	14.23	6.80	15.80	8.47	12.02	5.12
65-69	19.05	10.89	22.04	13.66	15.29	8.17
70-74	30.57	18.18	33.10	22.82	28.11	13.85
75-79	35.23	32.04	51.72	40.64	20.51	24.78
80-84	44.33	57.28	65.93	70.18	26.79	47.14
85 and over	85.00	129.08	112.68	129.53	70.31	128.82
