# Supplementary material for: Cause of death and potentially avoidable deaths in Australian adults with intellectual disability using retrospective linked data
Source: BMJ Open. 2017 Feb 2;7(2):e013489. doi: 10.1136/bmjopen-2016-013489 (PMC5306525; doi:10.1136/bmjopen-2016-013489)
Supplement: Supplementary table 2 [file bmjopen-2016-013489supp_table2.pdf]

Supplementary Table 2: Age-specific potentially avoidable death rate per 1000 persons, in people with and without ID

Age group	ID ABS Convention	ID Revised	Comparison
20-24	0.40	0.40	0.32
25-29	0.77	0.88	0.35
30-34	0.75	1.12	0.46
35-39	0.83	0.89	0.55
40-44	0.99	1.18	0.73
45-49	1.24	1.59	1.01
50-54	2.11	2.76	1.43
55-59	2.71	3.21	2.03
60-64	5.17	5.98	3.16
65-69	4.84	6.13	5.15
70-74	15.63	16.99	9.00
